# Neural correlates with individual differences in temporal prediction during auditory-motor synchronization

**DOI:** 10.1093/texcom/tgac014

**Published:** 2022-04-07

**Authors:** Kohei Miyata, Tetsuya Yamamoto, Masaki Fukunaga, Sho Sugawara, Norihiro Sadato

**Affiliations:** 1 Department of Life Sciences, Graduate School of Arts and Sciences, The University of Tokyo, 3-8-1 Komaba, Meguro, Tokyo 153-8902, Japan; 2 Department of System Neuroscience, National Institute for Physiological Sciences, 38 Nishigonaka, Myodaiji, Okazaki, Aichi 444-8585, Japan

**Keywords:** 7T, dorsal premotor cortex, fMRI, sensorimotor synchronization, temporal prediction

## Abstract

Temporal prediction ability is vital for movement synchronization with external rhythmic stimuli (sensorimotor synchronization); however, little is known regarding individual variations in temporal prediction ability and its neural correlates. We determined the underlying neural correlates of temporal prediction and individual variations during auditory-motor synchronization. We hypothesized that the non-primary motor cortices, such as the premotor cortex and supplementary motor area, are the key brain regions that correlate individual variations in prediction ability. Functional magnetic resonance imaging (7T) was performed for 18 healthy volunteers who tapped to 3 types of auditory metronome beats: isochronous, tempo change, and random. The prediction ability was evaluated using prediction/tracking ratios that were computed based on cross-correlations between tap timing and pacing events. Participants with a higher prediction/tracking ratio (i.e. stronger predictive tendency) tapped to metronome beats more accurately and precisely. The prediction/tracking ratio was positively correlated with the activity in the bilateral dorsal premotor cortex (PMd), suggesting that the bilateral PMd explains the individual variation in prediction ability. These results indicate that the PMd is involved in generating a model for temporal prediction of auditory rhythm patterns and its activity would reflect model accuracy, which is critical for accurate and precise sensorimotor synchronization.

## Introduction

Sensorimotor synchronization, which is the rhythmic coordination of action and perception, plays a pivotal role when dancing to music or playing music in an ensemble (Repp 2005; Levitin et al. 2018). Dancers express music physically by moving their bodies in coordination with the music being played, and musicians play instruments to harmonize the sounds produced by their co-performers in an ensemble (Keller et al. 2014). A beat, which is perceived as a pulse inferred from a rhythm occurring in equal temporal units, is the fundamental unit of measure of musical time (Levitin et al. 2018). Dancers and musicians perceive beats and coordinate their actions with the beats. Precise and accurate synchronization of movement with external events is crucial not only for musical performances but also for a wide variety of joint actions, such as marching and sports (Wing and Woodburn 1995).

One of the factors underlying successful synchronization is prediction ability. To synchronize with musical beats, one must extract temporal structures from ongoing events, generate an internal model of rhythm patterns, and predict the upcoming beat interval (Repp 2005). This prediction ability enables the planning and execution of sequential movements in a precisely timed manner; otherwise, the movement (e.g. finger taps) would lag behind the beat (i.e. reactive tapping). Thus, predicting the upcoming event interval is a basic prerequisite for sensorimotor synchronization. This assessment is supported by recent studies, which demonstrated that the temporal prediction ability of participants was associated with the stability of auditory-motor synchronization (Pecenka and Keller 2009, 2011; Mills et al. 2015). These studies focused on the ability of participants to tap in synchrony to pacing stimuli that contain gradual tempo changes. The tempo changes occur within a somewhat predictable range, as observed in music. The temporal prediction ability of participants was evaluated using the prediction/tracking ratio, which was computed based on the lag-0 and lag-1 cross-correlations between the inter-tap interval (ITI) and the inter-onset interval (IOI) of pacing ﻿stimuli. This ratio reflects the degree to which individuals’ taps predict or track the tempo changes. If individuals tend to predict tempo changes, then the lag-0 cross-correlation coefficient is high relative to the lag-1 cross-correlation coefficient (the ratio > 1) because prediction leads to a close match between the current ITI and IOI. On the other hand, a tendency to track is reflected in higher lag-1 than lag-0 cross-correlations (the ratio < 1) because the current ITI will most closely match the previous IOI when tracking (Repp 2002; Pecenka and Keller 2009, 2011; van der Steen and Keller 2013). Previous studies have suggested that prediction and tracking are not mutually exclusive and that individuals can engage in both behaviors simultaneously (Repp 2002; Rankin et al. 2009). Therefore, this ratio has been used to evaluate the prediction ability of temporal patterns. Using this index, studies have demonstrated that participants with higher prediction/tracking ratios tap more precisely to the beats (Pecenka and Keller 2009; Pecenka et al. 2013). This finding suggests that temporal prediction ability is related to sensorimotor synchronization skills.

Neuroimaging studies have investigated the neural basis of simple isochronous and more complex rhythm reproduction and synchronization (Rao et al. 1997; Chen et al. 2008a; Konoike et al. 2012). These studies implicate the involvement of several motor regions, such as the cerebellum, basal ganglia, premotor, and supplementary and pre-supplementary motor areas (SMA and pre-SMA, respectively). Pecenka et al. (2013) used functional magnetic resonance imaging (fMRI) to identify the neural correlates with temporal prediction associated with sensorimotor synchronization. They manipulated the temporal prediction tendencies of participants during auditory-motor synchronization by adding a visual n-back working-memory task. The prediction tendency estimated using the prediction/tracking ratios decreased with increasing the working-memory load. Using parametric analysis, the researchers found that brain activation in the distributed network covaried positively with the degree of prediction. These brain regions included the premotor cortex, SMA, and cerebellum.

However, little is known regarding the individual variations in temporal prediction ability. As aforementioned, previous studies have demonstrated that participants with higher prediction ability tapped more precisely to the beats (Pecenka and Keller 2009; Pecenka et al. 2013). Therefore, examining the individual variations in temporal prediction ability may provide important insights into behavioral and neural differences associated with sensorimotor synchronization skills. A previous study using an electroencephalogram (EEG) frequency-tagging approach suggested that this individual behavioral variation reflects the differences in brain activity (Nozaradan et al. 2016). Nozaradan et al. (2016) found that a stronger neural entrainment at the beat frequency was associated with superior temporal prediction abilities, which is indicative of a certain feature in brain activity related to prediction ability. EEG has high temporal resolution, which is optimal for assessing the temporal relationships between neural activity and behavior; however, it is also known to have low spatial resolution. Thus, the brain regions associated with this individual difference remain unknown. A candidate for this key brain region is the non-primary motor cortex, which includes the premotor cortex and SMA. These areas are known as higher-order motor areas involved in motor planning, motor preparation, and the sensory guidance of movement. Thus, these regions play an important role in holding serial sensory information and converting it to a movement program. Indeed, the non-primary motor cortex possesses reciprocal connections to the sensory cortex and has been considered a key link between multimodal sensory inputs and organized motor outputs (Reep et al. 1987; Barthas and Kwan 2017). Functional connections between the non-primary motor cortex and the auditory cortex have also been demonstrated in participants listening to various auditory rhythms (Chen et al. 2008b; Bengtsson et al. 2009; Grahn and Rowe 2009).

The aims of this study were to locate the brain regions related to auditory temporal prediction and to identify the key region responsible for individual variations in prediction ability during auditory-motor synchronization. An fMRI study was designed wherein the participants tapped to 3 sequence beats with differing temporal predictability. In the isochronous condition, the IOI was isochronous for 500 ms throughout a trial. The IOI changed following a triangle wave or randomly in a range from 400 to 600 ms during the tempo change or random conditions. The prediction ability was evaluated with the prediction/tracking ratios using sequence beats in the tempo change condition (Pecenka and Keller 2009). To estimate the synchronization skill, the mean asynchronies (the time difference between each metronome beat and the corresponding finger tap) and variance of asynchronies were calculated (Repp 2005; Pecenka and Keller 2009; Repp and Su 2013). The tendency of tapping to precede the beats by a few tens of milliseconds, known as the negative mean asynchrony, is lower in musicians than in non-musicians (Repp 2005). Among musicians, the negative mean asynchrony is lower among drummers than among amateur pianists (Krause et al. 2010). Moreover, the variance is generally lower for musically trained participants (Repp 2010; Miura et al. 2011). These findings indicate that the synchronization skill can be characterized by lower negative mean asynchrony and variance. Prediction-tracking ratios are known to correlate with the mean and variance of asynchronies (Pecenka and Keller 2009; Mills et al. 2015; Colley et al. 2018). In the present study, we hypothesized that the neural substrates involved in prediction show increased activation as the temporal predictability of pacing stimuli decreases. Moreover, they present different activation patterns based on an individual’s prediction ability.

## Materials and methods

### Participants

Eighteen right-handed healthy volunteers (10 female individuals; mean age, 21.4 years; range, 19–27 years) participated in the experiment. No participant reported a history of hearing impairment or any major medical, neurological, or psychiatric disorders. Participants had not received explicit musical training. This experiment conformed to the Declaration of Helsinki, and informed written consent was obtained from all participants. The study was approved by the Ethics Committee of the National Institute for Physiological Sciences. Our sample size was chosen based on previous studies (Pecenka et al. 2013; Nozaradan et al. 2016).

### Task and procedures

The experiment was performed in the magnetic resonance imaging (MRI) scanning room at the National Institute for Physiological Sciences (Okazaki, Aichi, Japan) that houses an ultra-high-field 7-Tesla MRI scanner. Upon arriving at the laboratory, the participants received an explanation regarding the purpose of this study, planned procedures, and potential risks and benefits of participation. After providing their informed consent, the participants filled a questionnaire regarding the MRI safety screening and musical background, as this has been shown to improve auditory-motor synchronization (Krause et al. 2010). Then, the participants wore an MRI gown.

While being in the MRI scanner, the participants were instructed to tap to auditory metronome beats with the right index finger to the best of their abilities while watching a fixation cross. There were 3 types of metronome beats: isochronous (I), tempo change (T), and random (R) (see Auditory and visual stimuli). ﻿In every run, each condition was presented 4 times in a randomized order. The participants completed 4 runs, resulting in a total of 48 trials (3 conditions × 4 repetitions × 4 runs). At the beginning of the experiment, each participant was given the opportunity to practice the task in each condition for a few minutes inside and outside the MRI scanner.

### Auditory and visual stimuli

**Fig. 1:**
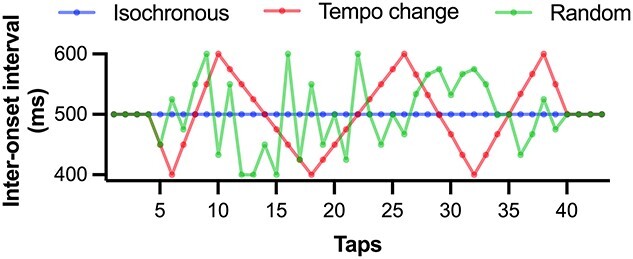
IOIs for the 3 experimental conditions. The IOIs for the isochronous, tempo change, and random conditions. The IOI was 500 ms throughout a trial in the isochronous condition. In the tempo change condition, the IOI lineally changed in the range of 400–600 ms, following a triangle wave. There were 6 tempo changes that occurred over the course of 4, 6, and 8 beats, ranging between 400 and 600 ms. In the random condition, the IOI of the tempo change condition was randomly ordered. The average IOI and number of taps for all 3 conditions was the same. IOI, inter-onset interval.

There were 3 types of metronome beats: isochronous (I), tempo change (T), and random (R; Fig. 1). The auditory metronome beats comprised woodblock sounds generated by a sampling pad (SPD-S, Roland Corporation, Hamamatsu, Shizuoka, Japan). The IOI was maintained at 500 ms throughout a trial in the isochronous condition. In the tempo change condition, the IOI was gradually changed from 400 to 600 ms following a triangle wave. The beats underwent 6 tempo changes, in which the IOI increased or decreased over a period of 4, 6, and 8 beats, with the changes ranging between 400 and 600 ms. To implement a jitter in the time point, at which the tempo changes commenced, the number of initial pacing beats varied from 4 to 7 beats. These initial beats were presented with an IOI of 500 ms, following which the tempo changed. The combination of tempo changes was randomly assigned under 2 constraints: (i) decreasing and increasing occurred alternatively, ensuring that the changes followed a triangle wave; and (ii) the tempo change would always start and end with a decrease in IOI. Therefore, one of the decreasing IOI changes was divided into 2 parts (500–400 ms and 600–500 ms) that were assigned as the first and last tempo changes. ﻿To ensure consistency in the number of tappings across conditions, isochronous tapping was required at the end of the trial that varied from 1 to 4 beats based on the initial isochronous beats. In the random condition, the IOIs of the tempo change condition were randomly ordered. The initial and final settings of the isochronous beats were the same as those in the tempo change condition. The average IOI for all 3 conditions was the same (i.e. 500 ms). Each stimulus sequence comprised 48 consecutive tones, including 4 initial tones as a ready cue. At the beginning of each trial, the ready cue was presented with 500 ms IOI and provided with a visual counting cue (i.e. 3, 2, 1, and Start), following which the fixation cross was presented. A beep signaled the end of the trial, and the trial duration was 25 s for all conditions.

### Experimental setup

The whole-brain functional images were acquired on a 7.0-T MR scanner (Magnetom 7T, Siemens Healthineers, Erlangen, Germany) with a 32-channel phased-array coil. The auditory and visual stimuli were generated using the Psychophysics Toolbox extensions (Brainard 1997; Pelli 1997; Kleiner et al. 2007) implemented in MATLAB 2017b (Mathworks Inc., Natick, MA, United States). The auditory metronome beats were delivered using in-ear headphones (KM-201W7, KOBATEL Corporation, Yokohama, Kanagawa, Japan) worn by the participants inside the scanner. The visual stimuli were projected onto a half-transparent screen that the participants in the scanner looked at through a mirror. The participants’ tapping responses were measured using an MRI-compatible button device (HHSC1x4-D, Current Designs Inc., Philadelphia, PA, United States).

### Behavioral data analysis

The first 3 taps in each trial were omitted from the analysis to discard the transient effects of changing from resting to tapping. The time differences in tapping were calculated for the beat that was closest to the tap. The mean and standard deviation (SD) of asynchronies within the trial were averaged across the trials.

The prediction ability was evaluated using the prediction/tracking ratios (Pecenka and Keller 2009; Pecenka et al. 2013), which were computed based on lag-0 and lag-1 cross-correlations between the IOI and ITI in the tempo change trials. The coefficient at lag-0 reflects how accurately a participant predicted the timing of the current IOI, whereas that at lag-1 indicates the participant’s ITI matching the timing of the previous IOI. The raw values of lag-0 and lag-1 are known to have limitations for the inference of prediction ability as each value has a theoretical lower limit based on the temporal structure of the sequence (i.e. the lag-1 autocorrelation of the sequence; Repp 2002; Pecenka et al. 2013). When prediction is optimal, the lag-1 correlation approaches the lag-1 autocorrelation because the ITI sequence is similar to the IOI sequence. When tracking is maximal, the lag-0 correlation approaches the lag-1 autocorrelation because the ITI echoes the IOI with a lag of 1 (Repp 2002). This lag-1 autocorrelation value differs depending on the timing patterns. In this study, a correction was needed because we randomly assigned the combination of tempo changes, resulting in different timing patterns among trials. As the correlations have the same lower limit (Repp 2002), we evaluated the prediction ability of participants by calculating the ratio of lag-0 to lag-1 following previous studies (Pecenka and Keller 2009; Pecenka et al. 2013). Thus, a prediction/tracking ratio > 1 indicates that the prediction is relatively stronger than the tracking, and the opposite is true when the ratio is < 1.

To calculate the prediction/tracking ratios, the number of ITIs was required to be the same as the number of IOIs. On average, 85.8% of the trials in the tempo change condition had equal numbers of ITIs and IOIs. In cases where the lengths were different, we performed interpolation following the methods of a previous study (Colley et al. 2018). In 8 out of 288 trials (2.8%) in the tempo change condition, because the participants missed >3 consecutive taps, the interpolation was unreliable and, therefore, excluded from the analysis.

### Statistical analysis of behavioral data

Separate one-way analyses of variance were performed on the mean asynchrony and the variability of asynchronies. The Greenhouse–Geisser correction was used in cases where Mauchly’s test of sphericity was significant. Pearson’s correlation coefficient was used to assess the relationship of the prediction/tracking ratios with the mean and SD of asynchronies. For all analyses, the statistical significance level was set at *P* < 0.05.

### Scanning procedure

The whole-brain functional images were acquired using a T2*-weighted echo planar imaging (EPI) sequence (repetition time = 1,000 ms; echo time = 22.2 ms; flip angle = 45°; acquired matrix = 130 × 130; field of view = 208 × 208 mm; slice thickness = 1.6 mm; 85 slices). A multi-band sequence was used to improve the acquisition speed (multi-band acceleration factor = 5; Moeller et al. 2010). Each run lasted ~8 min and provided 446 volumes. Although not included in this report, the structural images reflecting the white matter features were also acquired using diffusion-weighted imaging.

### Data analysis for MRI data

Preprocessing was performed using Human Connectome Project (HCP) Pipelines (v4.0.0-alpha.5; Glasser et al. 2013; Yamamoto et al. 2021). As described by Yamamoto et al. (2021), structural images were pre-acquired using a 3T MRI scanner (Magnetom Verio 3T; Siemens Healthineers), and functional images acquired using a 7T MRI scanner were pre-processed for gradient distortion correction, head motion correction, B0 distortion correction, normalization, and bias field correction.

To denoise the fMRI data, we performed a multirun independent component analysis (MR-ICA), which was implemented in the HCP Pipelines (v4.0.1; Glasser et al. 2018; Okamoto et al. 2020). MR-ICA can remove structured artifacts (Beckmann and Smith 2004; Smith et al. 2013; Griffanti et al. 2014; Salimi-Khorshidi et al. 2014). In MR-ICA, a set of EPI time-series are concatenated across runs to provide more data to the spatial ICA, resulting in better separation of signal and noise components. Before concatenating the time-series, the mean and variance of the time-series in each run were normalized across runs and high-pass temporal filtered (cut-off period = 2,000 s). Then, the normalized time-series were concatenated across the runs. Melodic ICA implemented in FSL (v6.0.1, Centre for Functional MRI of the Brain, Oxford University, UK) was run on the concatenated time-series data to produce component spatial maps and time-series. These components were manually classified into signal and noise categories based on the guidelines (Griffanti et al. 2017). The components classified as noise were non-aggressively regressed out from the EPI time-series (Smith et al. 2013). The denoised time-series was split back into the individual runs, and the spatial mean and variance profiles were restored to the individual runs.

**Fig. 2:**
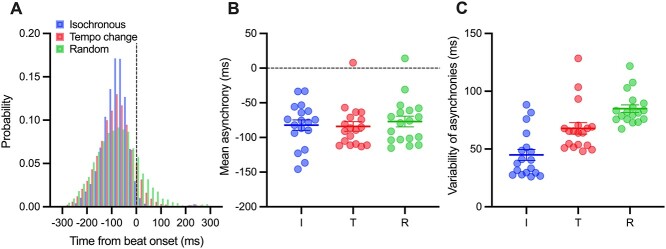
Distribution of the tapping onset time from beat onset for all participants A), the mean asynchrony B), and variability of asynchronies C) for the isochronous (blue), tempo change (red), and random conditions (green). In B) and C), each marker represents an individual participant. Vertical bars represent between-participant standard errors. I, isochronous condition; T, tempo change condition; and R, random condition.

The first-level statistical analysis was performed with statistical parametric mapping using SPM12 software (Wellcome Trust Centre for Neuroimaging, University College London, United Kingdom). The vectors containing the task block (duration = 25 s) were convolved with the canonical hemodynamic response function (HRF) to form the main regressors in the design matrix. The design matrix included 3 regressors of interest (I, isochronous; T, tempo change; and R, random), representing the metronome conditions. The model also included visual stimuli for instructions, which convolved with the HRF as a regressor of no interest. The data were estimated using the ordinary least squares method. The data were high-pass filtered (cut-off period = 128 s) to remove low-frequency signal drifts, and the AR (1) autocorrelation model was globally applied over the brain. To identify the brain areas associated with the predictability and prediction/tracking ratios, 3 contrast images of each condition (I, T, and R) and one contrast image of the tempo change condition with the isochronous condition (T > I) were constructed for each participant and used for the second-level analysis.

The second-level random effects analysis was performed to allow inferences across the participants using a flexible factorial design. A conjunction analysis was performed to explore the brain regions that were commonly engaged for each condition, irrespective of temporal predictability (I ∩ T ∩ R). To identify the neural correlates with predictability, neural activation in the random and tempo change conditions was compared with that in the isochronous condition (R > I and T > I, respectively). The isochronous condition was the most predictable, whereas the random condition was the most unpredictable in this experimental setting. The comparison of these 2 conditions indicates the contrast with the lowest predictability. The comparison between the tempo change and isochronous conditions had the next lowest predictability. To identify the regions associated with prediction ability, we examined parametric modulation of neural activity by the individual prediction/tracking ratios using the contrast image of the tempo change vs. isochronous conditions (T > I). This was because the prediction/tracking ratios could be calculated only in the tempo change condition﻿.

For all analyses, the resulting statistical values were organized by height thresholds with *P* < 0.001 (uncorrected), and a significant effect was reported when the volume of the cluster survived the false discovery rate at the cluster level (*P* < 0.05). The anatomical locations were determined using the SPM Anatomy Toolbox (Eickhoff et al. 2005, 2006, 2007), and the locations were verified using a paper atlas (Mai et al. 2015).

## Results

### Behavioral results

**Fig. 3:**
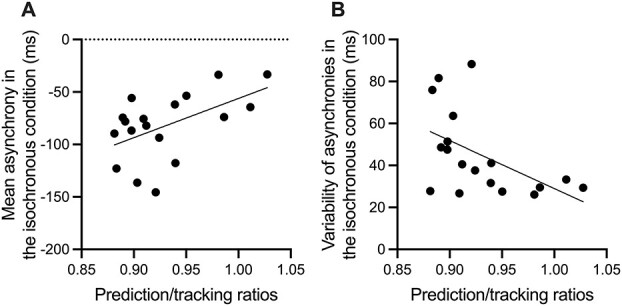
Scatter plot of the prediction/tracking ratios with A) mean asynchrony and B) variability of asynchronies in the isochronous condition. Each marker represents a participant. The trend line represents a linear regression line.

There were no significant difference in the mean asynchronies between the beats and the respective tap onsets (*F* [1.14, 19.32] = 0.67, *P* = 0.442). The variance or SD increased with the experimental control of predictability (*F* [1.96, 33.25] = 129.06, *P* < 0.001, ${\eta}_G^2$ = 0.45; Fig. 2). Participants with higher prediction/tracking ratios showed less asynchronous tapping (*r* [16] = 0.52, *P* = 0.026, *r^2^* = 0.27) and less variable tapping (*r* [16] = −0.51, *P* = 0.030, *r^2^* = 0.26) in the isochronous condition (Fig. 3).

### fMRI results

#### Conjunction analysis of all conditions

**Fig. 4:**
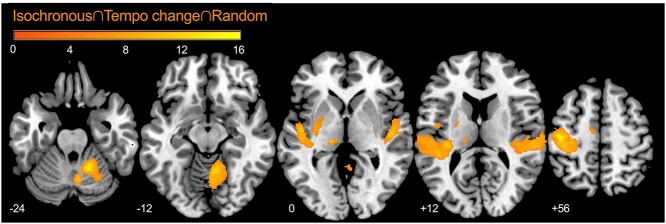
Conjunction analysis across the isochronous, tempo change, and random conditions. A significant cluster was overlaid on the MNI template image. Statistical thresholds were set at *P* < 0.001 (uncorrected) at the voxel level and at *P* < 0.05 (FDR-corrected) at the cluster level. MNI, Montreal Neurological Institute; FDR, false discovery rate.

The result of the conjunction analysis across conditions (I ∩ T ∩ R) revealed the brain regions involved in the synchronization of a tapping response to auditory rhythms irrespective of their predictability. These regions were the bilateral posterior superior temporal gyrus (STG), left primary sensorimotor cortex and thalamus, and right cerebellum lobule V (Fig. 4).

#### Neural responses to different degrees of predictability of pacing stimuli

**Fig. 5:**
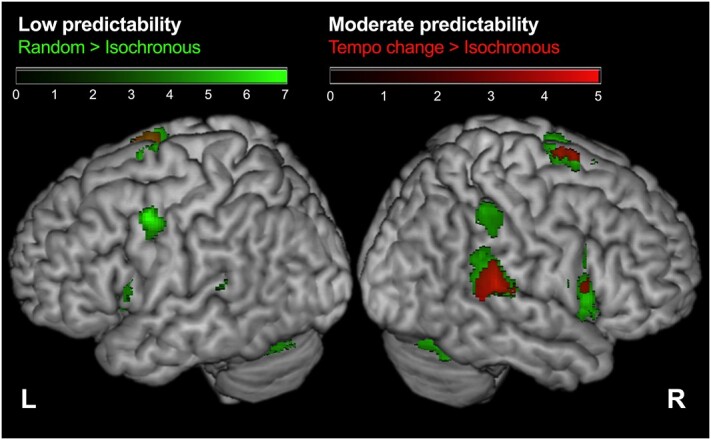
Neural responses to different degrees of predictability of pacing stimuli. A significant cluster was overlaid on a surface-rendered high-resolution anatomic MRI normalized to the MNI template. Statistical thresholds were set at *P* < 0.001 (uncorrected) at the voxel level and at *P* < 0.05 (FDR-corrected) at the cluster level. MRI, magnetic resonance imaging; MNI, Montreal Neurological Institute; and FDR, false discovery rate.

Figure 5 shows the neural substrates of the prediction of time-series of pacing stimuli, which are either easy (moderate predictability; Tempo change) or difficult (low predictability; Random). The activation levels of the right STG, inferior frontal gyrus (IFG), and SMA were higher in the tempo change condition than in the isochronous condition (T > I). In the random condition, significant activation was observed in the bilateral IFG, SMA, putamen, cerebellum, left ventral premotor cortex, and right primary sensorimotor cortex (R > I). The conjunction analysis between the tempo change and random conditions highlighted the left cerebellum, as well as the right STG, IFG, and SMA ([T > I] ∩ [R > I]). These are the regions involved in temporal prediction that were commonly found when we manipulated predictability.

The activation levels of the medial prefrontal cortex, bilateral precuneus, and left hippocampus were higher in the tempo change condition than in the random condition (T > R). In the opposite contrast, the right cerebellum showed higher activation (R > T).

#### Activations correlated with the prediction/tracking ratios

**Fig. 6:**
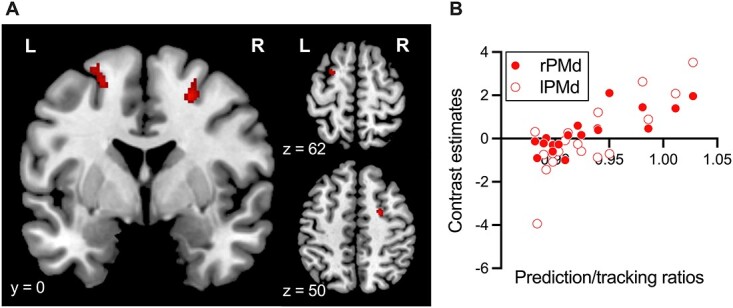
A statistical parametric map illustrating the cluster (red) that was significantly correlated with prediction/tracking ratios using the contrasts in tempo change (T > I). Significant clusters were overlaid on the MNI template image. Statistical thresholds were set at *P* < 0.001 (uncorrected) at the voxel level and at *P* < 0.05 (FDR-corrected) at the cluster level. The scatter plot demonstrates the correlation between contrast estimates at [23, −1, 50] (right PMd) and [−22, 2, 58] (left PMd) and the prediction/tracking ratios. MNI, Montreal Neurological Institute; FDR, false discovery rate; rPMd, the right dorsal premotor cortex; lPMd, the left dorsal premotor cortex.

Figure 6 shows the brain regions whose activities were positively correlated with the prediction/tracking ratios in the tempo change condition (T > I). The contrast estimates at these regions suggested that activation was stronger with higher prediction/tracking ratios. The regions included the bilateral dorsal premotor cortex (PMd). Thus, individuals with higher prediction/tracking ratios showed higher activity in the bilateral PMd.

## Discussion

The present study aimed to locate and identify the brain regions involved in prediction ability, which was quantified using the prediction/tracking ratios. These ratios are used as an index of prediction ability and reportedly correlate with tapping performance. Our behavioral results were consistent with those of previous studies demonstrating that the prediction/tracking ratios were correlated with the accuracy and precision of tapping to isochronous beats. Thus, participants with high prediction/tracking ratios (i.e. with a stronger predictive tendency) showed less asynchronous tapping and less variable tapping in the isochronous condition.

The novel finding of the present study was that the prediction/tracking ratios were correlated with the activity of the bilateral superior frontal sulcus, which likely corresponds to the rostral portion of the PMd (PMdr) based on connectivity-based parcellation (Tomassini et al. 2007). The PMd is structurally and functionally different from the ventral part of the premotor cortex (PMv; Wise et al. 1997; Hoshi and Tanji 2006). Nonhuman primate studies showed that PMd neurons are particularly active during a preparatory-motor-set period (Weinrich and Wise 1982; Wise 1985). In contrast, PMv neurons respond to somatosensory stimuli applied to either the face or the arm and to visual stimuli corresponding to peripersonal stimuli (Fogassi et al. 1996; Graziano et al. 1997). There is a functional gradient in the PMd (Hanakawa et al. 2002). Nonhuman primate studies showed that the PMdr closely interconnects with the prefrontal cortex rather than M1 (Barbas and Pandya 1987; Lu et al. 1994) and lacks direct projection to the spinal cord (He et al. 1993). The PMdr is involved in the sensory aspects of sensorimotor integration more than the caudal counterpart (Weinrich and Wise 1982; Johnson et al. 1996; Shen and Alexander 1997), independent of immediate movement, and more closely related to the function of the prefrontal cortex. Such a rostrocaudal gradient of the PMd functions likely exists in humans too (Rizzolatti et al. 1998; Geyer et al. 2000). The PMdr of the human is located anterior to the superior precentral sulcus and dissociate from the caudal part by the vertical anterior-commissural plane (Deiber et al., 1991; Rizzolatti et al. 1998).

The PMd has been reported to be involved in auditory-motor synchronization and is emphasized as an important node that facilitates auditory-motor interactions in the context of rhythm (Zatorre et al. 2007; Chen et al. 2009; Pollok et al. 2009). The functional connectivity between the PMd and STG (i.e. auditory regions) was found to increase on tapping to auditory rhythms (Chen et al. 2008a; Pollok et al. 2009). Moreover, PMd activity appears to be sensitive to the temporal complexity of auditory rhythms. Functional connectivity between the STG and PMd was a function of metric saliency (i.e. the contrast in sound amplitude between accented and unaccented tones) when participants tapped in synchrony to isochronous rhythms (Chen et al. 2006). A meta-analysis of 34 neuroimaging studies on auditory-motor synchronization demonstrated that the activation pattern of the premotor cortex for temporal and ordinal complexity was similar to that of the cerebellum. Moreover, at a higher temporal complexity, the premotor cortex showed greater activation than the SMA and the sensorimotor cortex (Janata and Grafton 2003). These findings suggest that the PMd is involved in the selection of temporally organized movements based on a higher-order metrical structure derived from the auditory stimulus.

Previous brain stimulation studies have also emphasized on the importance of the PMd during auditory-motor synchronization (Pollok et al. 2008; Giovannelli et al. 2014). On-line repetitive transcranial magnetic stimulation (rTMS), which leads to a suppression of cortical excitability, on the left PMd results in increased asynchrony and variability of tapping (Pollok et al. 2008). In contrast, off-line rTMS results in increased asynchrony when applied to the right PMd, but not when applied to the left PMd and the SMA (Giovannelli et al. 2014). Moreover, this effect was observed when tapping to structured (metrical) rhythmic sequences but not when tapping to isochronous nor unstructured rhythmic sequences (Giovannelli et al. 2014). Neither cathodal nor anodal transcranial direct current stimulation over the left PMd affected auditory-motor synchronization with respect to the isochronous rhythm (Pollok et al. 2017). These findings suggest that the PMd is crucial for accurate tapping when the rhythmic sequence was a complex. In addition, these reports support the role of PMd in the selection of temporally organized movements based on a higher-order metrical structure. The current study extends previous findings by demonstrating that PMd involved in individual variation in prediction ability, which is critical for accurate tapping to complex rhythmic sequences.

Concerning perception, PMd activation might be involved in auditory imagery in the working memory. Keller (2012) proposed that musicians predict their co-performers’ ongoing action outcomes by using internal simulation processes to generate anticipatory auditory images of the other performers’ sounds (i.e. auditory imagery; Pecenka et al. 2013). This assumption is supported by findings from a previous study, which revealed a positive correlation between the prediction/tracking ratios and the acuity of auditory imagery for pitch (Pecenka and Keller 2009). Auditory imagery preserves the structural and temporal properties of auditory stimuli, thus, relying on the working memory (Hubbard 2010). The prediction of the timing of the next beat might be based on the most recent series of IOIs; hence, one needs to store the most recent series of beats in the working memory. Such auditory imagery in the working memory would be essential for the selection of temporally organized movements based on a higher-order metrical structure. Therefore, individual differences in PMd activity might be related to the accuracy of auditory imagery in the working memory.

To depict the neural correlates of auditory temporal prediction, we utilized the contrast of random—isochronous, and tempo change—isochronous. The predictability of auditory pacing stimuli was high in the isochronous condition and low in the random condition. The variability of tapping increased in the experimental conditions, suggesting a decrease in its predictability (Fig. 2c). As the mean asynchrony was negative, the depicted neural areas may represent the prediction process, which includes both internal model formation and its comparison with the cued signals to generate prediction error. Assuming prediction processes are, at least partly, common between the tempo change and random conditions, we applied the conjunction analysis, showing the activation of the right IFG, STG, and SMA, and the left cerebellum (Fig. 5). This finding replicated the previous study (Pecenka et al. 2013). Although the IFG, SMA, and cerebellum are traditionally associated with motor control, the STG is associated with the auditory process. A previous study demonstrated the attention-related modulation of activity in the temporal cortex (Grady et al. 1997). Thus, the activity in the STG might reflect strong attention to auditory stimuli.

We did not observe the activation of the PMd in the neural responses to different degrees of predictability of pacing stimuli. As discussed so far, the PMd may mediate auditory-motor interactions by extracting higher-order information from the preceded auditory stimuli, thus, aiding to generate internal models for temporal prediction of rhythm patterns distributed in the IFG, STG, SMA, and the cerebellum, which in turn coordinate motor processes to execute actions at an appropriate time (Chen et al. 2006, 2008a, 2008b; Pecenka et al. 2013). The PMd showed activation only in response to individual differences in prediction accuracy (Fig. 6). Therefore, the individual variance in PMd activity represents the working memory recruitment required for better temporal prediction.

The conjunction analysis across conditions provided the core network of auditory-motor synchronization, such as perceiving the metronome sounds, generating actions (e.g. the right index finger movement), and integrating the perception and action. The network consists of the bilateral posterior STG, left primary sensorimotor cortex and thalamus, and right cerebellum lobule V. These findings are consistent with those of previous studies (Rao et al. 1997; Repp 2005; Repp and Su 2013; Levitin et al. 2018); thus, the cerebello–thalamo–cortical network plays an executive role when tapping to auditory beats.

In our study, negative mean asynchrony was also found in the random condition. When the next beat is unpredictable, a participant usually has no choice but to react and respond to it, thus, resulting in a positive mean asynchrony. In this study, we asked participants to tap as accurately as possible but did not tell them to change their tapping strategy to reactive tapping, as this would mean performing a different task. Furthermore, although different IOIs were presented randomly in the random condition, the IOIs were nearly 500 ms (mean IOI, 500 ms; range 400–600 ms). Therefore, even under the random condition, the participants might have tried to perform predictive tapping based on the 500 ms IOI, resulting in negative mean asynchrony with high variability. One might speculate that the observed negative mean asynchrony was attributed to the method of calculating asynchrony. As there is no way to ascertain which beat each participant intended to tap to, asynchrony was calculated for the beat that was closest to the tap. However, if the participants reactively responded to the tones one before, the positive mean asynchrony would fall around the range of 400 ms, as the mean ITI was 500 ms. This explanation is unlikely because the mean reaction time is the range of 200–300 ms. Furthermore, the negative mean asynchrony of isochronous, temporal change and random conditions are all around 100 ms, thus it is unlikely only the random condition reflected the different strategy, that is, reactive tapping instead of predictive tapping.

Our study had certain limitations. In this study, the prediction/tracking ratios were used to assess prediction ability of the upcoming beat interval during auditory-motor synchronization. As this index is based on the ratios between lag-0 and lag-1 cross-correlations (prediction tendency and tracking tendency), it cannot be applied to tapping in the isochronous (where the IOIs are constant) or in the random condition (where tracking behavior would not occur, as the previous IOI is not related to the subsequent IOI). Individual variation in prediction ability should also exist in these 2 conditions, but the prediction/tracking ratio model cannot evaluate this. A new measure of prediction ability that is independent of the temporal structure of tapping is required to clarify its neural basis in future studies. In addition to prediction, temporal adaptation (reactive error correction) is an important factor for auditory-motor synchronization (van der Steen and Keller 2013; van der Steen et al. 2015). Prediction and adaptation are interwoven; therefore, further studies are warranted from the perspective of both prediction and adaptation to understand behavioral and neural differences associated with sensorimotor synchronization skills. Finally, the present study was limited by a relatively small sample size (*N* = 18) to investigate individual variation. Nonetheless, this study revealed the neural correlates with individual differences in temporal prediction during auditory-motor synchronization. This might have been attributed to the fact that we performed a 7T MRI examination. The MRI systems operating at field strengths greater than 3T (i.e. ultra-high filed at 7T and above) provide significantly increased signal-to-noise ratio and sensitivity to small blood oxygenation level dependent signal (Torrisi et al. 2018; Cai et al. 2021). A previous study demonstrated that a single run of 7T MRI has 4 times the model predictive power of 3T MRI (Cai et al. 2021). Therefore, despite the relatively small sample size in the present study, the effect size and statistical power were expected to be high. Although our findings might explain only a small part of individual variation in temporal prediction, they suggest fruitful avenues for future studies.

In conclusion, the ability to accurately synchronize to musical rhythm is dependent on both the predictability of the temporal structure of the sequence, and the individual prediction ability. The current study demonstrated that the SMA, right IFG, STG, and left cerebellum are related to temporal structure prediction and that the bilateral PMd explains the individual variation in prediction ability. These findings are applicable to domains beyond those of music and dance and can account for the coordination between perception and action that plays an important role in our daily life.
